# jvenn: an interactive Venn diagram viewer

**DOI:** 10.1186/1471-2105-15-293

**Published:** 2014-08-29

**Authors:** Philippe Bardou, Jérôme Mariette, Frédéric Escudié, Christophe Djemiel, Christophe Klopp

**Affiliations:** Plate-forme bio-informatique Genotoul/MIA-T, INRA, Borde Rouge, 31326 Castanet-Tolosan, France; Plate-forme SIGENAE/GenPhySE, INRA, Borde Rouge, 31326 Castanet-Tolosan, France

**Keywords:** Venn, Edward-Venn, Vizualisation, jquery, JavaScript

## Abstract

**Background:**

Venn diagrams are commonly used to display list comparison. In biology, they are widely used to show the differences between gene lists originating from different differential analyses, for instance. They thus allow the comparison between different experimental conditions or between different methods. However, when the number of input lists exceeds four, the diagram becomes difficult to read. Alternative layouts and dynamic display features can improve its use and its readability.

**Results:**

jvenn is a new JavaScript library. It processes lists and produces Venn diagrams. It handles up to six input lists and presents results using classical or Edwards-Venn layouts. User interactions can be controlled and customized. Finally, jvenn can easily be embeded in a web page, allowing to have dynamic Venn diagrams.

**Conclusions:**

jvenn is an open source component for web environments helping scientists to analyze their data. The library package, which comes with full documentation and an example, is freely available at http://bioinfo.genotoul.fr/jvenn.

## Background

High-throughput biology has led to an increasing number of data, with more and more complex experimental designs. The analysis of these data often produces biological identifier lists, including gene names or OTU (Operational Taxonomic Unit), obtained from different methods (for differential analysis) or from different experimental conditions. Venn diagrams [1] are a common visualization chart, which allows to spot shared and unshared identifiers providing an insight on lists similarities.

**Figure 1:**
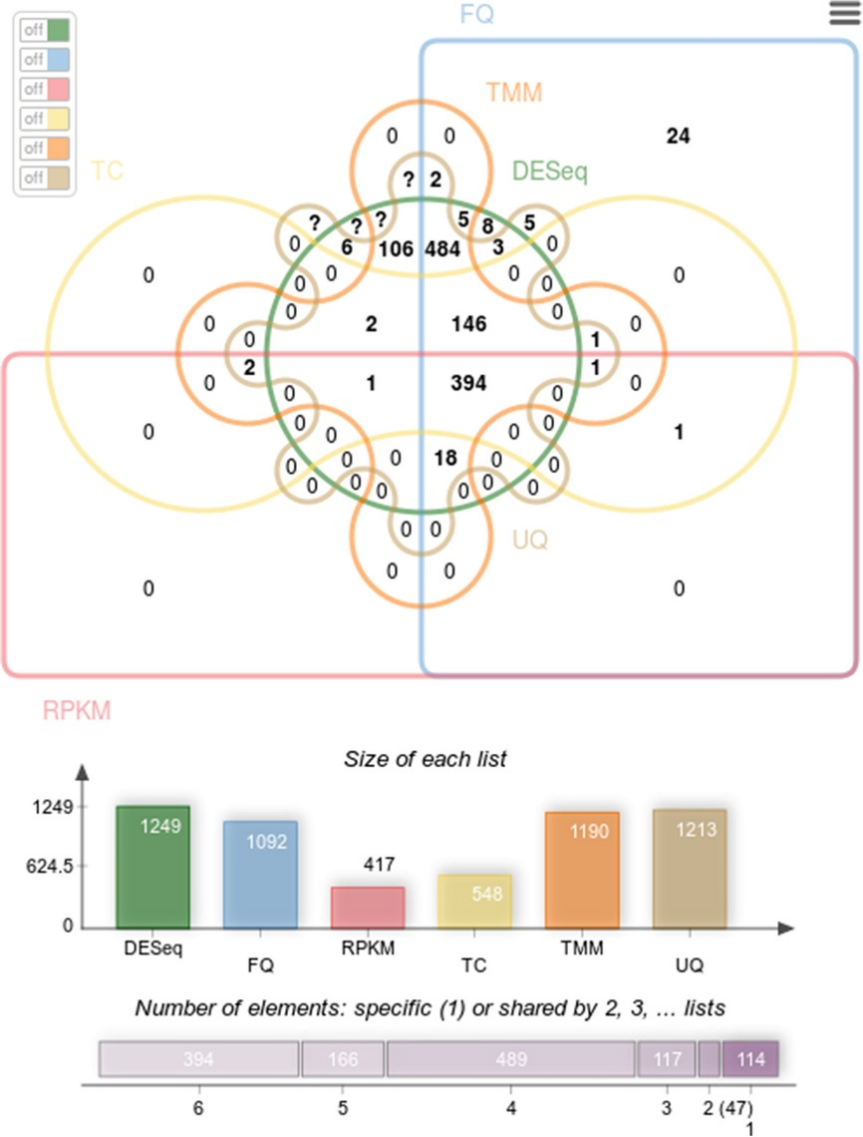
**A six lists Edwards-Venn diagram.** This Venn diagram displays overlaps between six different biological samples. The icon, located on the top-right, allows users to download the diagram as a PNG file. The middle-right switch button panel allows to activate or dis-activate lists to access a specific intersection count. Charts showing the list size and intersection size repartition located underneath the diagram.

In a Venn diagram, each list is presented by a transparent shape. Shape overlaps contain the elements shared between lists or more often the corresponding counts. In proportional Venn diagrams, the size of a shape is proportional to the number of elements of the corresponding list or of the corresponding lists intersection. Venn diagrams with up to four lists are easy to read and understand but Venn diagrams with more than four lists, are much harder to interpret. To solve this problem, the Edwards-Venn [2] representation introduces new shapes providing a clearer view, shown in the example of Figure 1.

Many Venn diagram software packages are already available. The first six lines of Table 1 present the main packages with their main features (maximum number of input lists, input data formats, Venn diagram layouts, application types and output formats). The table gives insight on several aspects of the Venn diagram production and highlights that, up to now, no web application handled up to six lists. VENNTURE [3] is the only application able to produce such diagrams but it only implements Edwards layout and runs only under MS-Windows OS, producing static MS-PowerPoint and MS-Excel files. Proportional Venn diagrams can only display a very limited number of lists, three at maximum. The only feature available in other software which is not in jvenn is the proportional diagram. This is justified by the fact that jvenn was designed to display up to six lists and that proportional diagram is not suited to visualize more than three lists.

**Table 1:** **Features of a subset of already available software packages, and jvenn**

Application	Maximum number of	Layouts	Application type	Proportionality	Input data formats	Output formats
	input lists					
VENNTURE [3]	6	Edwards	Stand-alone	No	Lists	Powerpoint
						and Excel
VennDiagram [4]	5	Classical	R package	No	Lists	R object
						and TIFF
BioVenn [5]	3	Classical	web application	Yes	Lists	SVG and PNG
venny [6]	4	Classical	web application	No	Lists	PNG
Canvasxpress [7]	4	Classical	JavaScript library	No	Intersection	JavaScript
					counts	canvas
Google	3	Classical	JavaScript library	Yes	Lists	PNG
Chart API [8]						
jvenn	6	Classical	web application	No	Lists,	Interactive
		and Edwards	and JavaScript		intersection	diagram,
			library		counts and	PNG and CSV
					Count lists	

Hereafter we introduce jvenn, a JavaScript library, developed as a jQuery plug-in [9], including many features easing diagram production and enhancing their readability. In particular, jvenn can handle up to 6 lists, is a dynamic tool and implements both proportional and Edwards layouts. The library has already been used and cited in two scientific publications [10, 11]. It is already embedded in different web applications such as nG6 [12], RNAbrowse [13] and WallProtDB [14].

## Implementation

This section presents the main features of the jvenn library, including the kind of inputs it accepts, the different types of charts it displays, the types of the outputs and how it can be integrated in websites or directly used on our example web page.

### Inputs

The jvenn library accepts three different input formats : “Lists”, “Intersection counts” and “Count lists”. Examples are presented in Table 2, where the different lists are “sample1” and “sample2”, the elements of the different lists are given in the fields “data”. For “Intersection counts”, the lists are given a label (“A” or “B”) which is used to make the correspondence between the list and its count. Finally, “Count lists” provide a count number for each element of a list. Hence, with “Count lists” the figures presented in the diagram correspond to the sums of counts of all elements shared between lists. they can be particularly useful to present OTU read counts [11]. For “Lists” and “Count lists”, jvenn computes the intersection counts and displays the chart. For “intersection counts”, the intersection counts is provided by the user.

**Table 2:** **Available input formats**

Format	Example
	series: [{
	name: ’sample1’,
	data: [~Otu1~, ~Otu2~, ~Otu3~, ~Otu4~, ~Otu5~, ~Otu6~, ~Otu7~]
Lists	}, {
	name: ’sample2’,
	data: [~Otu1~, ~Otu2~, ~Otu5~, ~Otu7~, ~Otu8~, ~Otu9~]
	}]
	series: [{
	name: {A: ’sample 1’, B: ’sample 2’},
Intersection counts	data: {A: [~Otu3~, ~Otu4~, ~Otu6~], B: [~Otu8~, ~Otu9~], AB: [~Otu1~,
	~Otu2~, ~Otu5~, ~Otu7~]} }],
	values: {A: 3, B: 2, AB: 4}
	series: [{
	name: ’sample1’,
	data: [~Otu1~, ~Otu2~, ~Otu3~, ~Otu4~, ~Otu5~, ~Otu6~, ~Otu7~],
	values: [5, 15, 250, 20, 23, 58, 89]
Count lists	}, {
	name: ’sample2’,
	data: [~Otu1~, ~Otu2~, ~Otu5~, ~Otu7~, ~Otu8~, ~Otu9~],
	values: [90, 300, 10, 2, 45, 9]
	}]

### Display features

**Figure 2:**
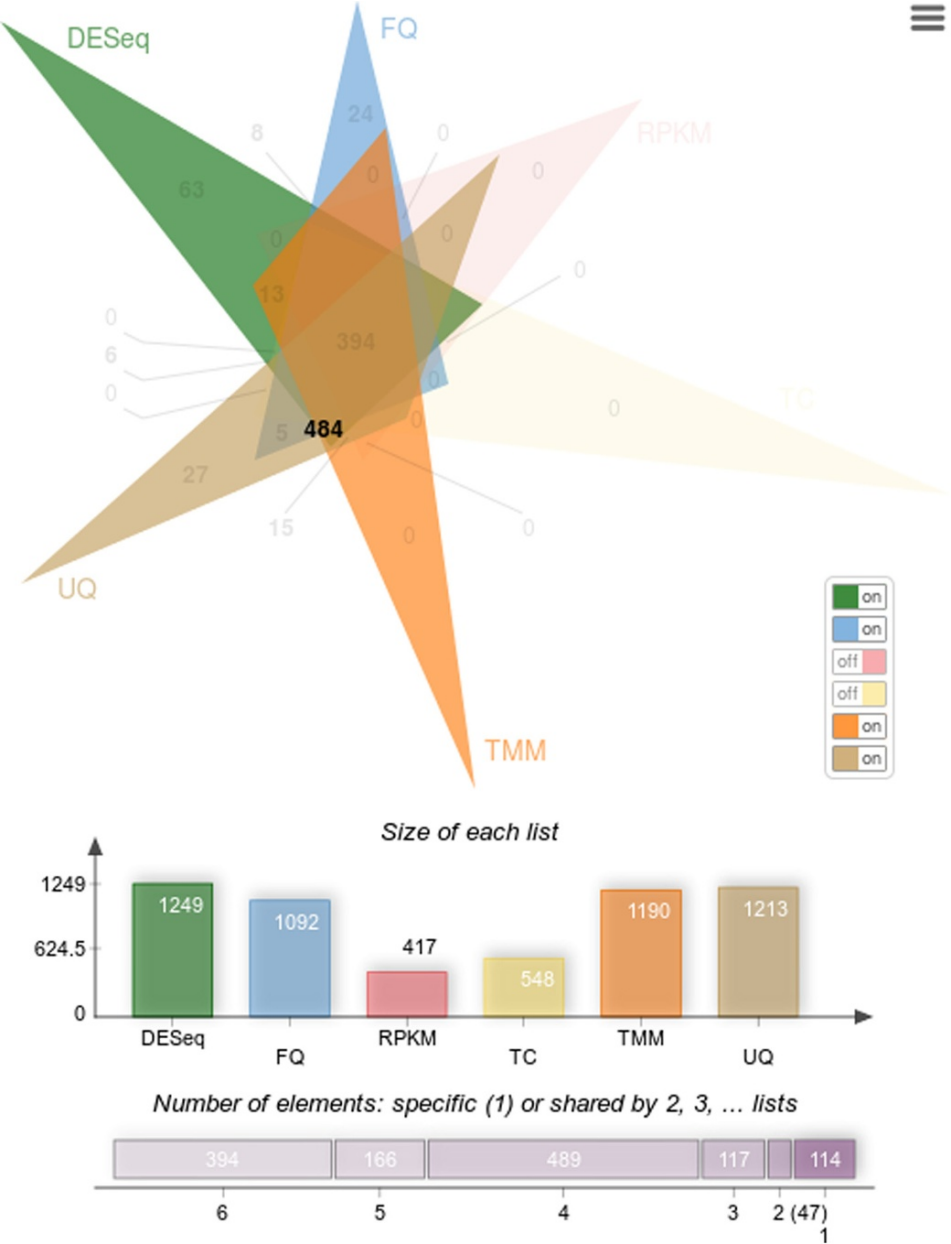
**A six lists classic Venn diagram.** On mouse over a figure, the shape corresponding to the lists involved in the intersection are highlighted and the other ones faded out. In this example, the user pointed the intersection between DESeq, FQ, UQ and TMM which contains 484 different genes.

Venn diagrams are commonly used to present up to six lists but for six lists, the intersection areas obtained when using a proportional layout are often too small to display the figures.To display five or six lists diagrams, in a user-friendly manner, jvenn implements several features. First, the layout can be switched between the standard layout and the Edwards-Venn layout (Figure 1) which gives a clearer graphical representation for six lists diagrams. To enhance the figure’s readability for the classical six lists Venn chart, some count values are not shown and some are display outside the chart, using lines to line the count to its corresponding area. However, this is still not enough to show all figures. Therefore, a switch button panel (right side of Figure 2) was added. It enables to switch on and off the different lists and to display the corresponding intersection counts. When the number of characters of the intersection count exceeds the available space to display it, the value is substituted by a question mark. When the mouse is mouved over this question mark, the value pops-up. To emphasize the list involved in an intersection area, jvenn highlights the intersection shapes when mouse is moved over, fading the others out.

The extra charts presented under the Venn diagram ease the verification and comparison of multiple lists. The list size graph allows users to check the homogeneity of the input list sizes. The intersection size graph can be used to compare the compactness of multiple Venn diagrams.

Scientists are usually interested in extracting identifier lists for some intersections, therefore, jvenn implements a one-click function which retrieves the names of the corresponding sets and the identifiers. To find an identifier, one can use a dynamic search box. The shapes containing the matching identifiers are highlighted when using this tool.

### Outputs

jvenn display is based on a JavaScript canvas object that allows for PNG export. The intersection table can also be downloaded as a CSV file. This file contains a header line with the diagram area labels and, in column, the identifiers of the elements contained in the area.

### Integration

jvenn allows programmers having only moderate JavaScript experiences to embed Venn diagrams in a web page without dependency. It has been designed following the examples of jbrowse [15], Cytoscape-Web [16], and jHeatmap [17]. The integration documentation is included in the software package which can be downloaded from http://bioinfo.genotoul.fr/jvenn.

### Web application

**Figure 3:**
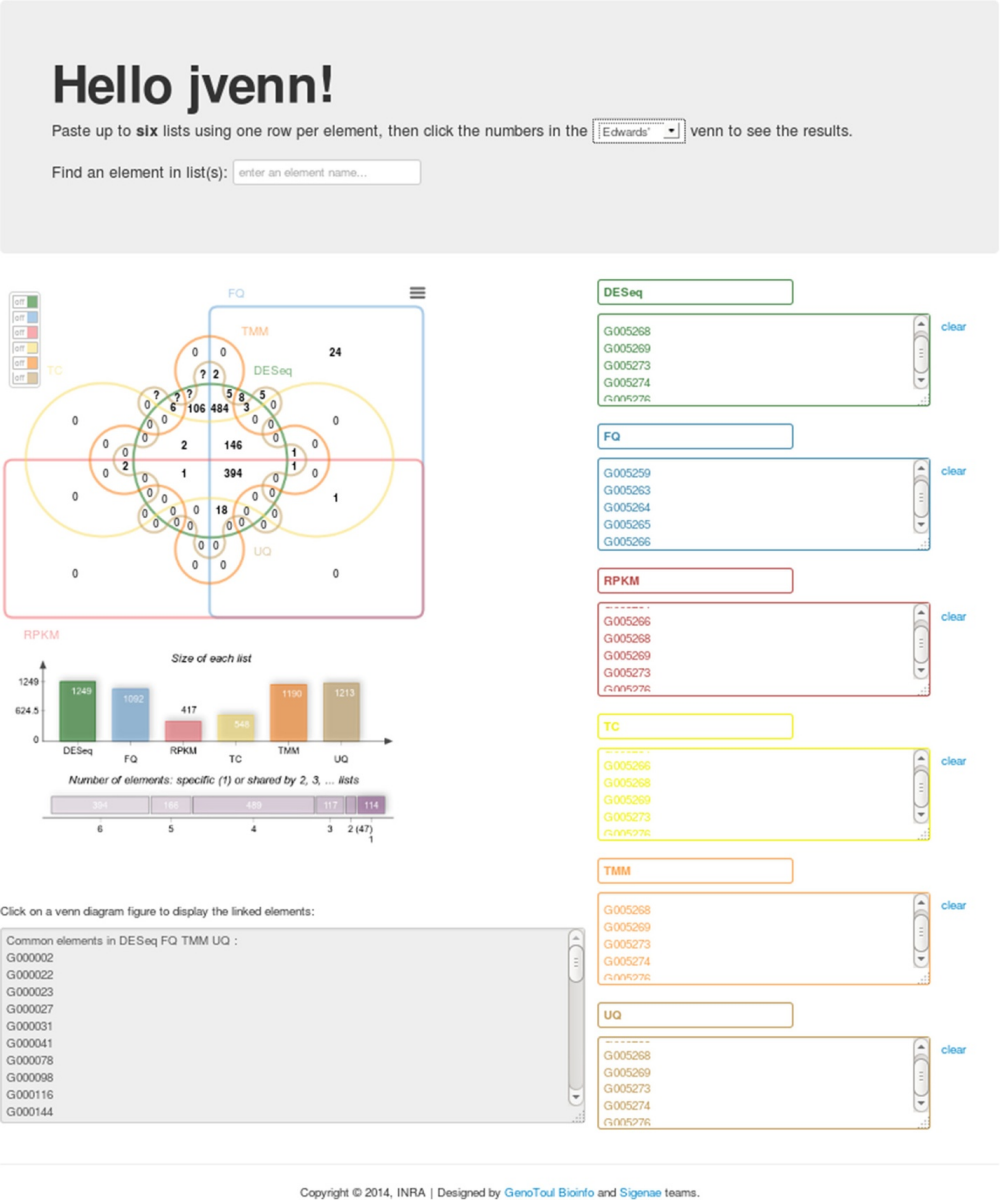
**jvenn web application.** The running version of jvenn accessible at http://bioinfo.genotoul.fr/jvenn/example.html. This one allows the user to set all jvenn main features. The layout can be changed from classical to Edwards, a search box is accessible, the switch button panel and the exporting button are available and the statistical charts are displayed. On the right of the page, each of the six textareas can be filled with a list of elements (one per line). If the same item is given multiple times, this one will be considered as unique. The list labels can also be customized using the text field on the top of each textarea.

jvenn can also be directly used as a web application, which is available at http://bioinfo.genotoul.fr/jvenn/example.html(Figure 3). jvenn’s web application performances depend on the client browser. Using the current version on a standard Linux computer (one cpu, four GB of RAM), it displays a six lists diagram of 10,000 identifiers in two seconds.

## Results

M.A. Dillies and colleagues [18] have compared seven methods for normalization and search of differentially expressed genes in RNASeq data. This study is designed to provide a set of best practices to help biologists with their data processing. Table 2 of the cited article is the contingency table of the differentially expressed genes obtained from the seven methods, where counts in the table correspond to the intersection of two lists obtained from two different methods. The raw data table, kindly provided by the team, contains 5,277 lines and seven columns. The columns correspond to the different methods presented in the “Differential expression analysis” section of their article. The data in the table was filtered (*p*<0.05) to retrieve the gene name lists corresponding to each method. As, jvenn handles only six list at most, six out of the seven lists were selected for further processing: we left out the median normalization method because, for one hand, this method is very similar to several other methods (as shown in the article) and, for the other hand, we believe that median is a poor estimate of the sequencing length, which is the bias that normalization methods try to correct. The lists were uploaded to the jvenn application and a Venn diagram was obtained, using an Edwards layout, which is shown in Figure 1.

The same analysis was performed with VENNTURE, the only other tool able to generate a six list Edwards Venn diagram. First, the software package was installed on a computer running under MS-Windows OS. The six gene lists were loaded in an MS-Excel spreadsheet and VENNTURE was run using the spreadsheet as input generating a static MS-PowerPoint file containing the diagram and a MS-Excel file with all the intersection contents.

## Discussion

The lists overlaps, as produced by jvenn, are given in Figure 1 (Edwards layout) and Figure 2 (standard layout). The highest counts are located in central areas of the graph, showing that the corresponding methods share large portions of gene lists. The jvenn statistics show that the different methods produce gene lists with very different sizes (minimum 417 - maximum 1,249) and that most of the genes are shared between methods: 1,069 genes out of 1,347 are common between at least four methods. In a very intuitive manner, the chart also points out that the results are strongly consensual since there are many zeros in the peripheral areas. Only a few genes (114) are specific to one list only (24 for FQ, 27 for UQ and 63 for DESeq, which appears to be the less restrictive method, as shown in the barplot below the Venn diagram, and also the most different from the others). Genes that are in two lists only are also very few (47: 13 for DESeq and TMM, 5 for UQ and FQ, 15 for TMM and UQ, 8 for FQ and DESeq and 6 for DESeq and UQ). Note that all these numbers are easily read from the chart and that the strong consensus between the lists is also clearly shown from the upper side figure “Number of elements: specific or shared by several lists”). Such findings are not easily shown using only contingency tables.The largest count over all lists overlaps is found to be 484, which is the number of genes found to be differential by DESeq, TMM, UQ and FQ. As shown in Figure 3, this list is very easily retrieved from the web application in one click only, providing the biologist with a large list of very consensual list to study.On the other hand, if the biologist is interested in one specific gene, this gene can easily be tracked using the search box at the top side of Figure 3. As no specific gene is of interest in the seminal work, we simply picked out one of the 5,277 genes randomly (G002562) and used it in the search box. It was found to be part of the five genes specific to FQ and UQ.

Making the same analysis with VENNTURE is also possible but a bit harder: the 484 genes shared by DESeq, TMM, UQ and FQ can be found easily in the intersection spreadsheet outputed by VENNTURE but the diagram did not allow to search for gene G002562. Thus, this gene has to be found using MS-Excel text search in the intersection spreadsheet, which is less handy than a dynamic and interactive search. Moreover, the additional statistics are not provided by the tool.

## Conclusion

jvenn enables to compare up to six lists and updates the diagram automatically when modifying the lists content. Compared to VENNTURE it does not need any local installation of a new program and it gives access to a dynamic diagram providing simple tools to extract gene lists and perform searches. jvenn’s statistics charts give a simple and quick overview of the sizes of the different lists and of their overlaps. It permits to compare different Venn diagrams. These features are not available in the VENNTURE software package.

For biologists using different techniques in their experiment or in their statistical analysis, jvenn enables to quickly extract the shared identifiers. When comparing different methods applied to extract differentially expressed genes, these features ease the analysis.

Thanks to its numerous features, dynamic behavior and graphical layout quality, jvenn can be efficiently used in many cases to compare different sets of results and easily extract shared elements. Being a simple JavaScript plug-in allows developers to embed it in any web environment.

## Availability and requirements

-  **Project name:** jvenn
-  **Project home page:**http://bioinfo.genotoul.fr/jvenn
-  **Project demo site:**http://bioinfo.genotoul.fr/jvenn/example.html
-  **Operating system(s):** Platform independent
-  **Programming Language:** Javascript
-  **Other requirements:** Web browser
-  **License:** GNU GPL
-  **Any restrictions to use by non-academics:** GNU GPL
